# Supplementary material for: ER-associated degradation regulates Alzheimer’s amyloid pathology and memory function by modulating γ-secretase activity
Source: Nat Commun. 2017 Nov 13;8:1472. doi: 10.1038/s41467-017-01799-4 (PMC5684335; doi:10.1038/s41467-017-01799-4)
Supplement: Supplementary file 1 — Supplementary Information [file 41467_2017_1799_MOESM1_ESM.pdf]

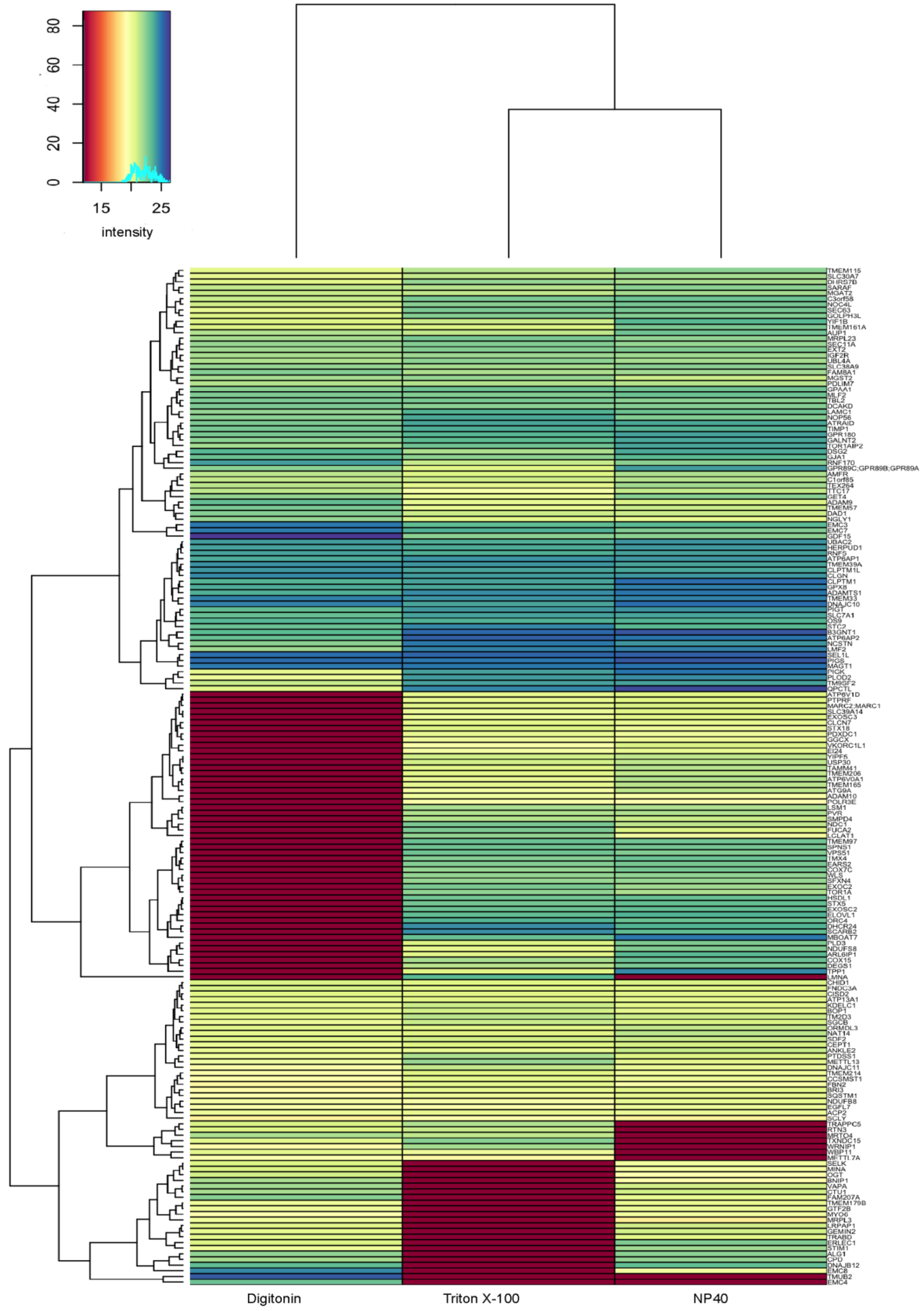

Supplementary Figure 1. Heatmap of 180 membralin interacting proteins identified using three different lysis buffers

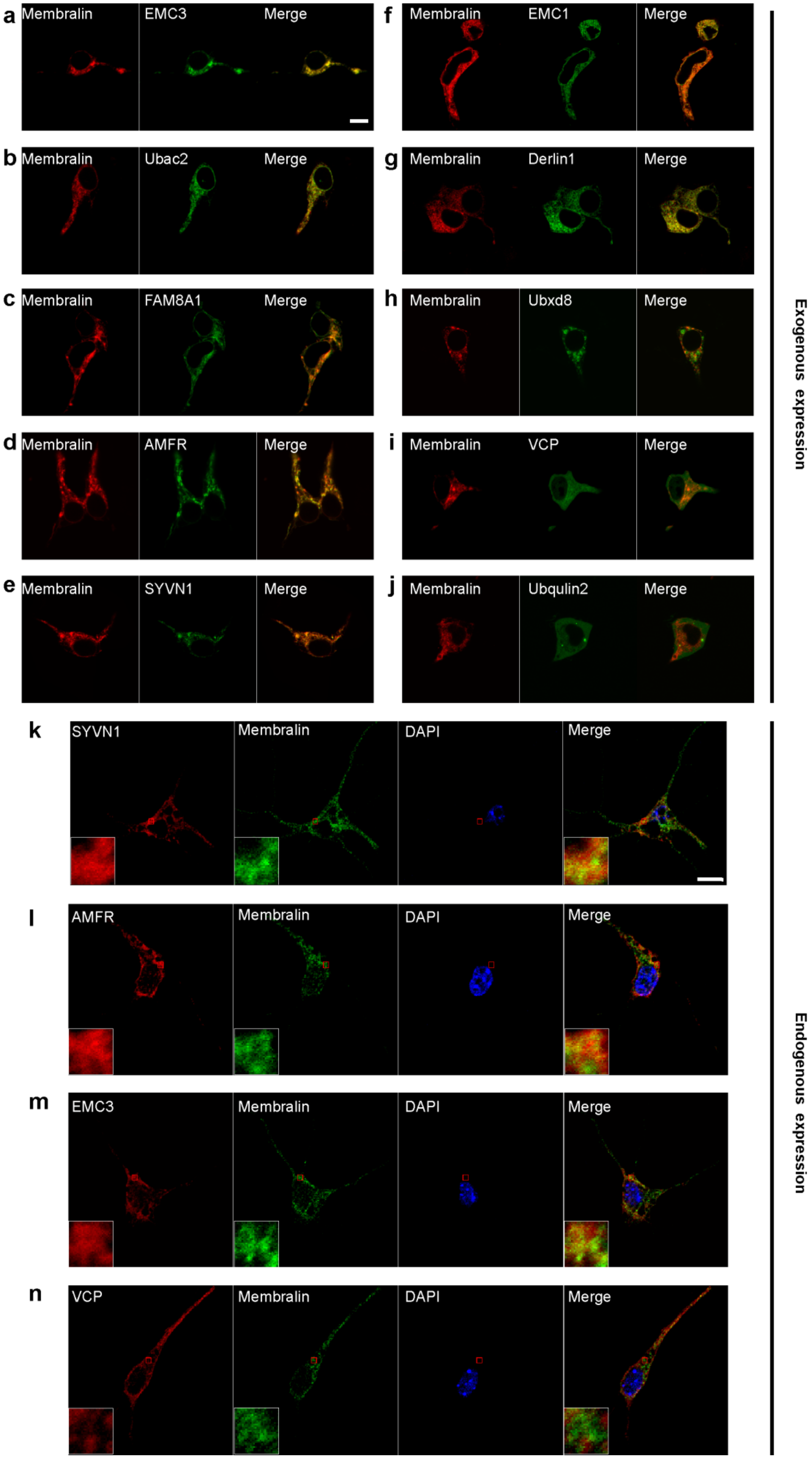

a
Membralin
EMC3
Merge
b
Membralin
Ubac2
Merge
c
Membralin
FAM8A1
Merge
d
Membralin
AMFR
Merge
e
Membralin
SYVN1
Merge
f
Membralin
EMC1
Merge
g
Membralin
Derlin1
Merge
h
Membralin
Ubxd8
Merge
i
Membralin
VCP
Merge
j
Membralin
Ubqulin2
Merge
Exogenous expression
k
SYVN1
Membralin
DAPI
Merge
l
AMFR
Membralin
DAPI
Merge
m
EMC3
Membralin
DAPI
Merge
n
VCP
Membralin
DAPI
Merge
Endogenous expression

Supplementary Figure 2. Colocalization of exogenous and endogenous membralin with candidate interacting proteins Confocal images of HEK293T cells co-transfected with membralin-mCherry and: (**a**) EMC3-EGFP, (**b**) Ubac2-EGFP, (**c**) FAM8A1-EGFP, (**d**) AMFR-EGFP, (**e**) SYVN1-EGFP, (**f**) EMC1-EGFP, (**g**) Derlin1-EGFP, (**h**) Ubxd8-EGFP, (**i**) VCP-EGFP, or (**j**) Ubquilin2-EGFP. Scale bar, 10 µm. Primary neurons at DIV 7 are co-stained with anti-membralin (green) and: (**k**) anti-SYVN1 (red), (**l**) anti-AMFR (red), (**m**) anti-EMC3 (red), (**n**) anti-VCP (red). Scale bar, 10 µm.

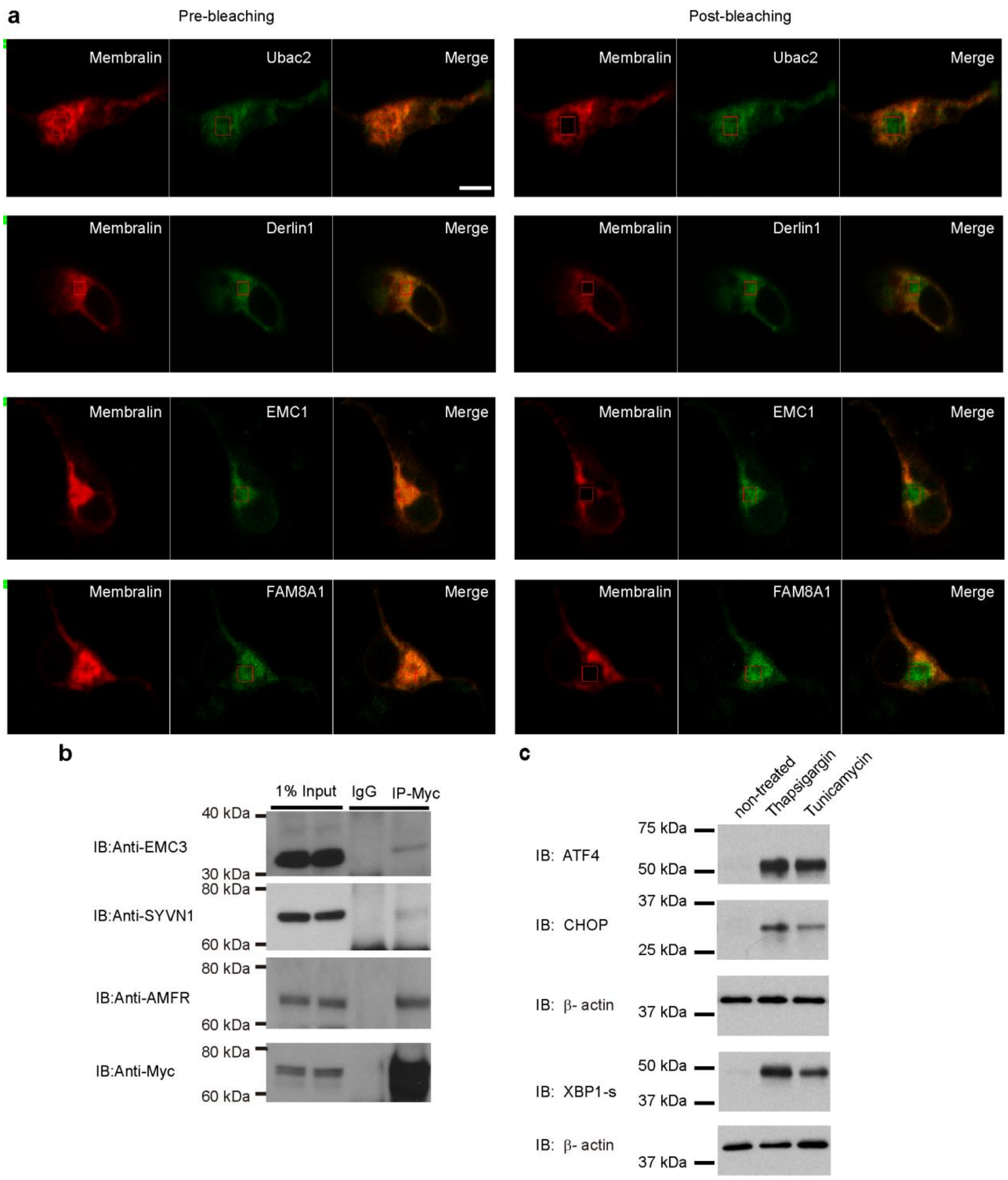

Supplementary Figure 3. Confirming potential membralin interactions by FRET and co-IP analysis (**a**) FRET images (pre- and post-bleaching) of cells co-transfected with membralin-mCherry and either Ubac2-EGFP, Derlin1-EGFP, EMC1-EGFP or FAM8A1-EGFP. Scale bar, 10 µm. (**b**) Co-IP analysis of Myc-membralin precipitates with endogenous EMC3, SYVN1, or AMFR. (**c**) The UPR pathway was activated upon ER stress induced by thapsigargain (500 nM) or tunicamycin (5 µg/ml) in the N2a cells, as indicated by elevated ATF4, CHOP, and XBP1-s expression.

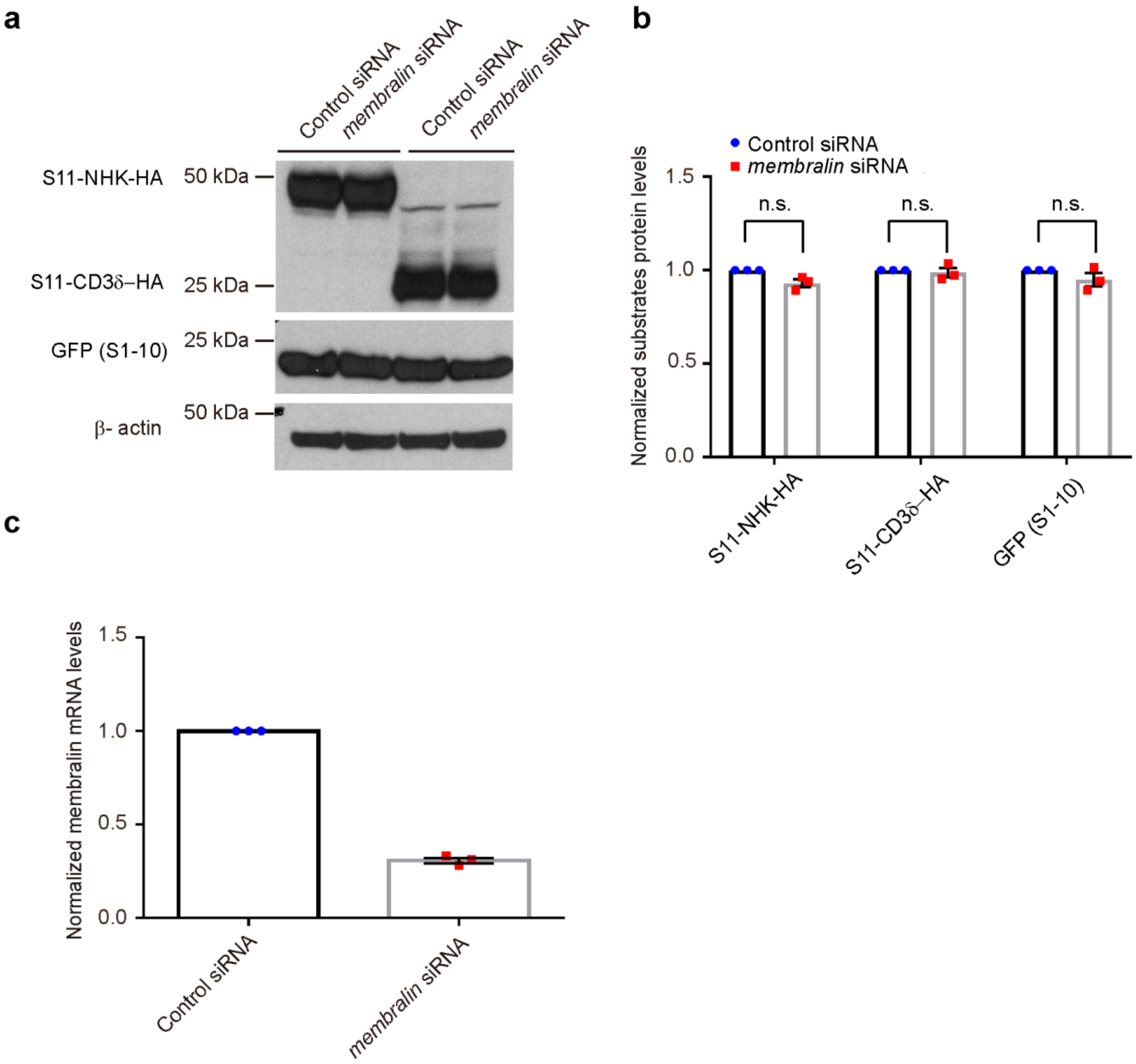

Supplementary Figure 4. S11-NHK-HA and S11-CD3δ-HA protein expression levels with control and *membralin* siRNA transfection (**a**) Western blot analysis of S11-NHK-HA and S11-CD3δ-HA expression using an anti-HA antibody, and GFP (S1-10) protein expression levels in control siRNA and *membralin* siRNA. (**b**) Quantification of S11-NHK-HA, S11-CD3δ-HA and GFP (S1-10) protein expression levels. Data represent mean ± s.e.m. from 3 independent experiments, one-way ANOVA, n.s., not significant. (**c**) Efficiency of membralin knockdown in HEK293T cells. Data represent mean ± s.e.m. from 3 independent experiments.

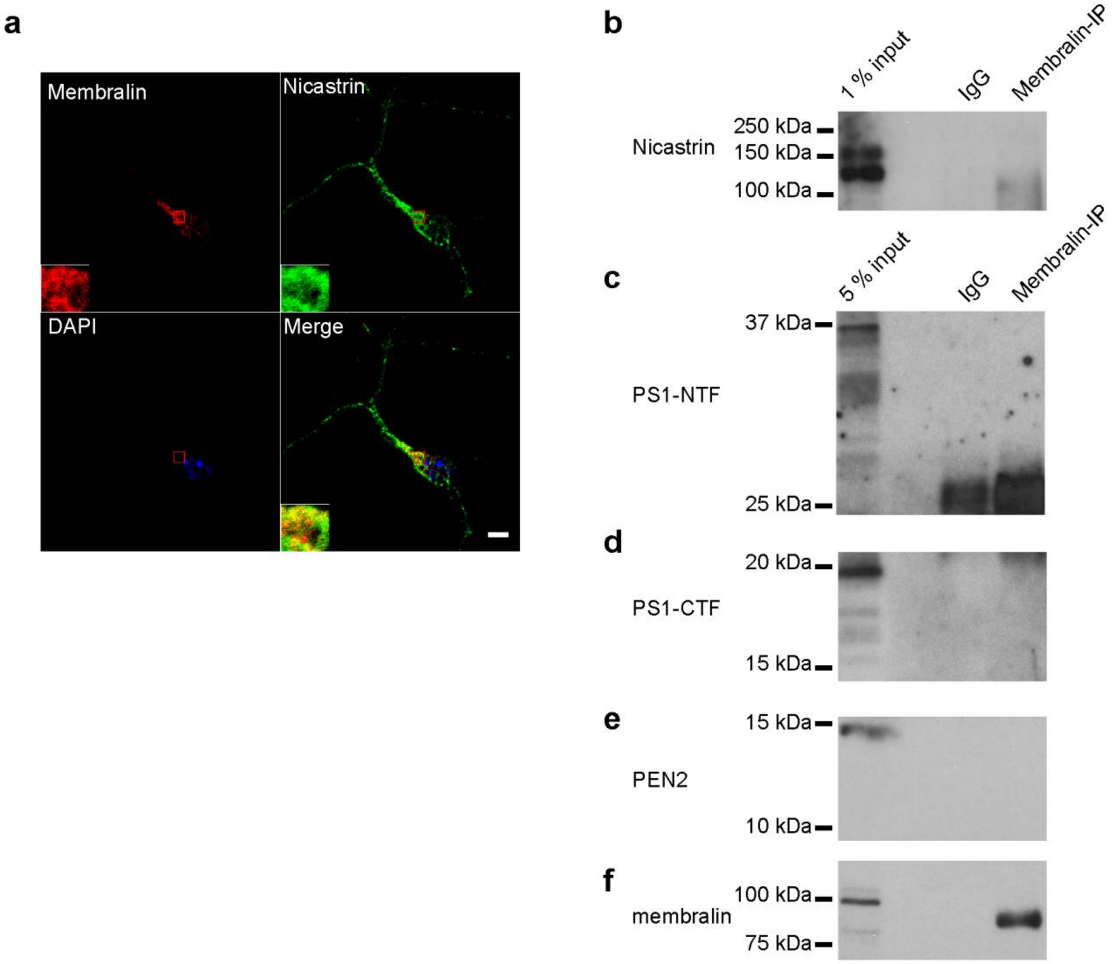

Supplementary Figure 5. Co-localization of endogenous membralin and nicastrin in cultured primary mouse neurons (**a**) Primary neurons at DIV 7 were stained with anti-membralin (red) and anti-nicastrin (green) antibodies. Scale bar, 5 µm. (**b-f**) Co-IP analysis of membralin interactions with different γ-secretase components: nicastrin, PS1-NTF, PS1-CTF, and PEN2, respectively using membrane enriched fractions purified from N2a cells.

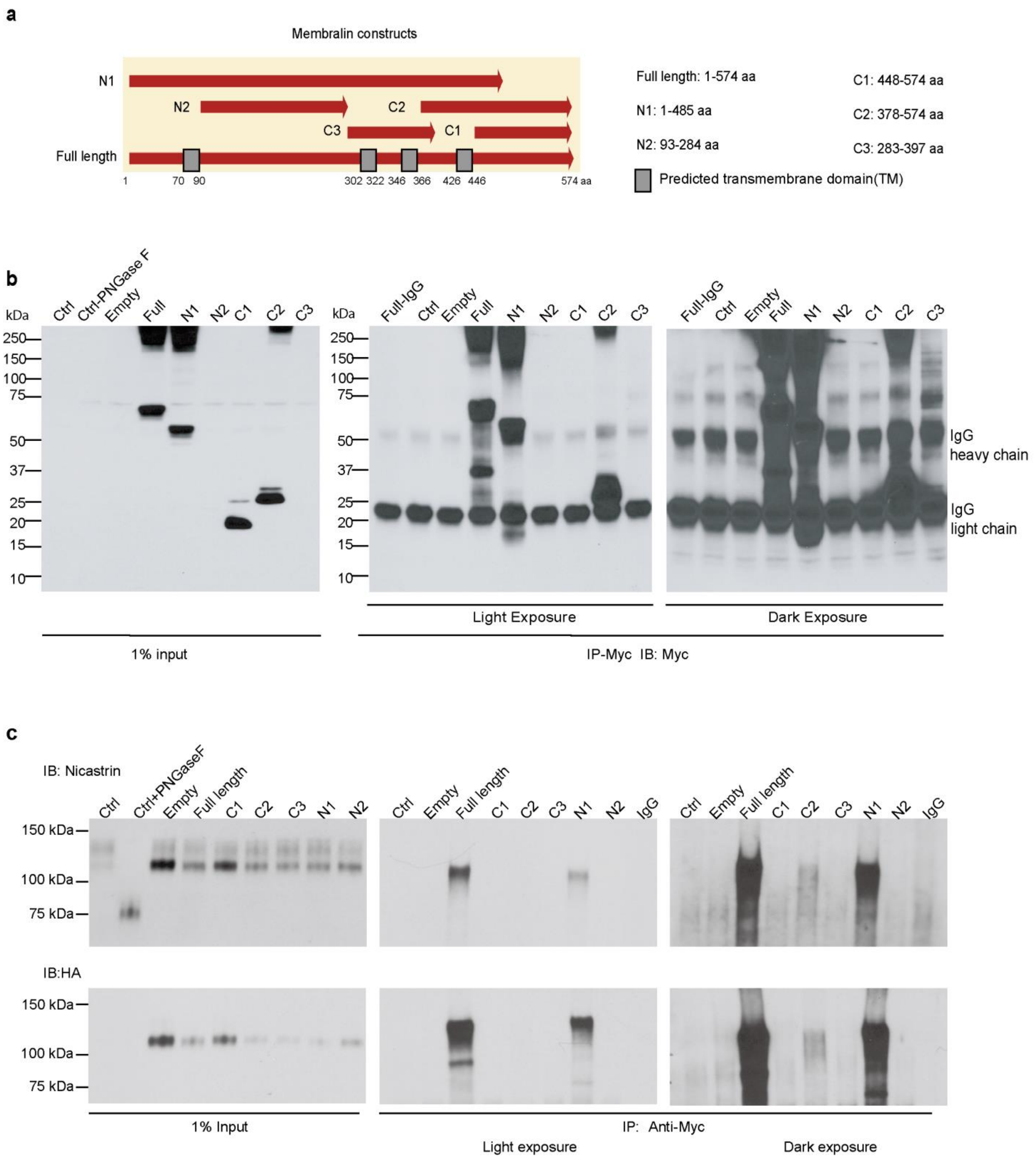

Supplementary Figure 6. Mapping domain interactions in the membralin/nicastrin complex (**a**) Membralin deletion constructs design. Membralin comprises four predicted transmembrane domains (gray boxes). (**b**) Expression of Myc-membralin constructs as detected by western blot and IP. (**c**) C2 and N1 membralin constructs can be precipitated with nicastrin or HA antibodies in HEK293T cell lysates co-expressing HA-nicastrin and various Myc-membralin constructs.

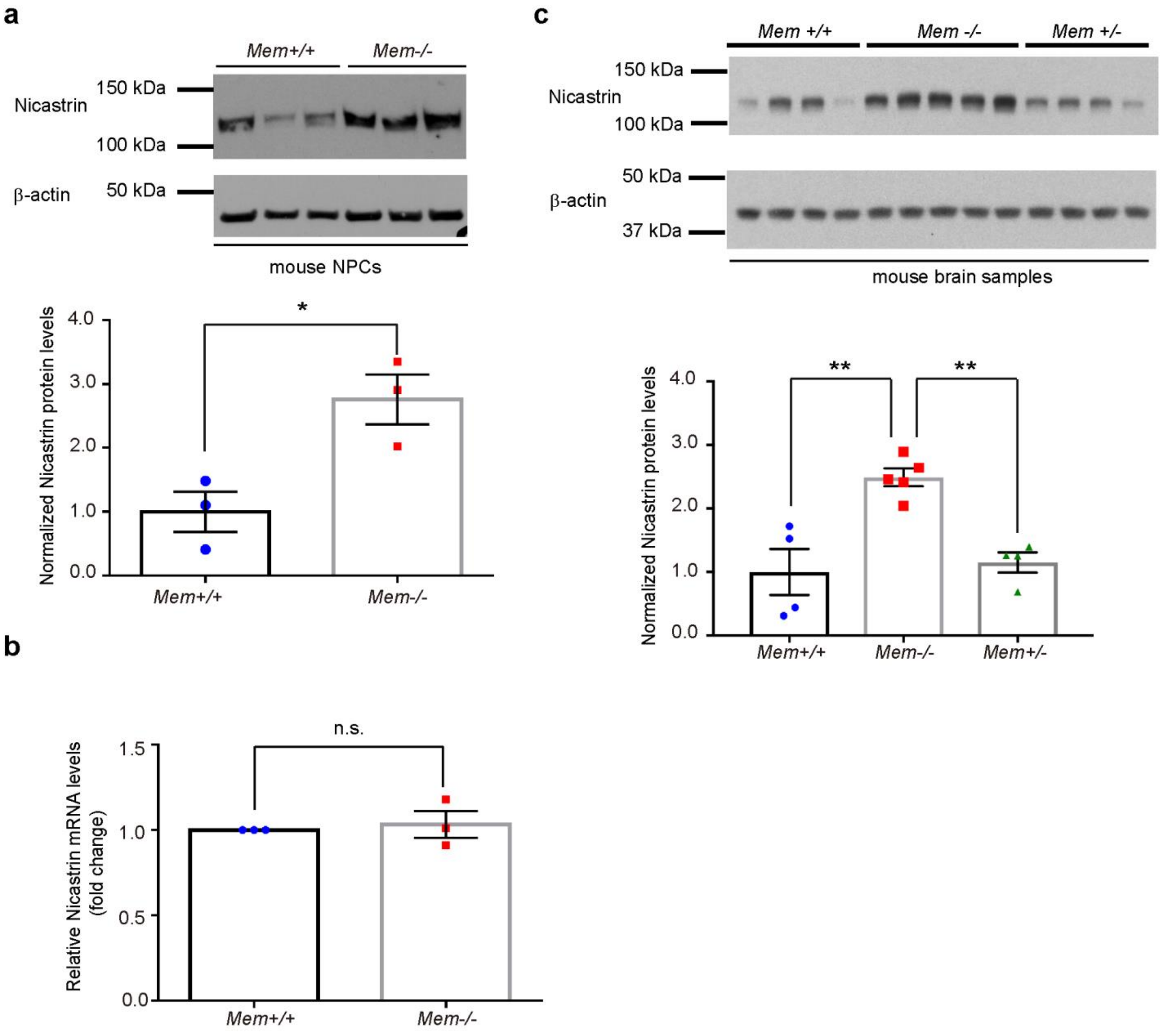

Supplementary Figure 7. Nicastrin mRNA and protein levels in *Mem+/+* and *Mem-/-* NPCs and brain tissues (**a**) Western blot analysis and quantification of nicastrin protein levels in *Mem+/+* and *Mem-/-* mouse NPCs. (**b**) Real-time qPCR analysis of nicastrin mRNA levels in *Mem+/+* and *Mem-/-* mouse NPCs. Data represent mean ± s.e.m. from 3 independent experiments, unpaired *t*-test, * $P < 0.05$, n.s., not significant. (**c**) Western blot analysis and quantification of nicastrin protein levels in *Mem+/+* (n = 4 mice), *Mem-/-* (*n* = 5 mice), *Mem +/-* (*n* = 4 mice) brain samples. Data represent mean ± s.e.m. One way ANOVA, ** $P < 0.01$.

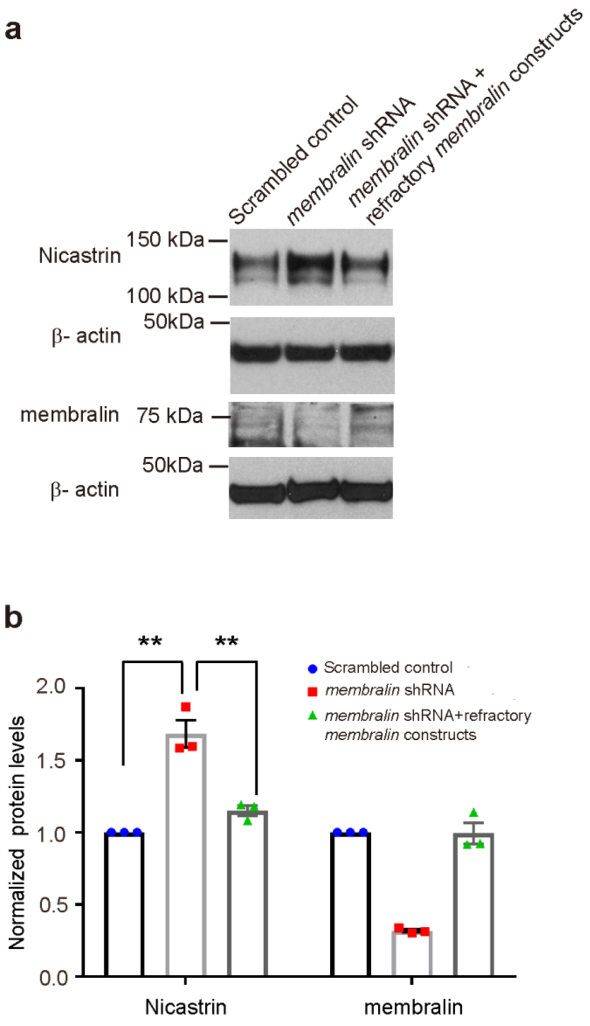

Supplementary Figure 8. *Membralin* shRNA expression rescue analysis to determine off-target effects associated with *membralin* shRNAs (**a**) Western blot analysis of nicastrin and membralin protein levels in scrambled control or *membralin* shRNA transfected, and *membralin* shRNA/refractory mouse *membralin* co-transfected N2a cells. (**b**) Quantification of nicastrin and membralin protein levels in scrambled control, *membralin* shRNA and *membralin* shRNA/refractory mouse *membralin* constructs co-expressed in N2a cells. Data represent mean ± s.e.m. from 3 independent experiments, one-way ANOVA, ** $P < 0.01$.

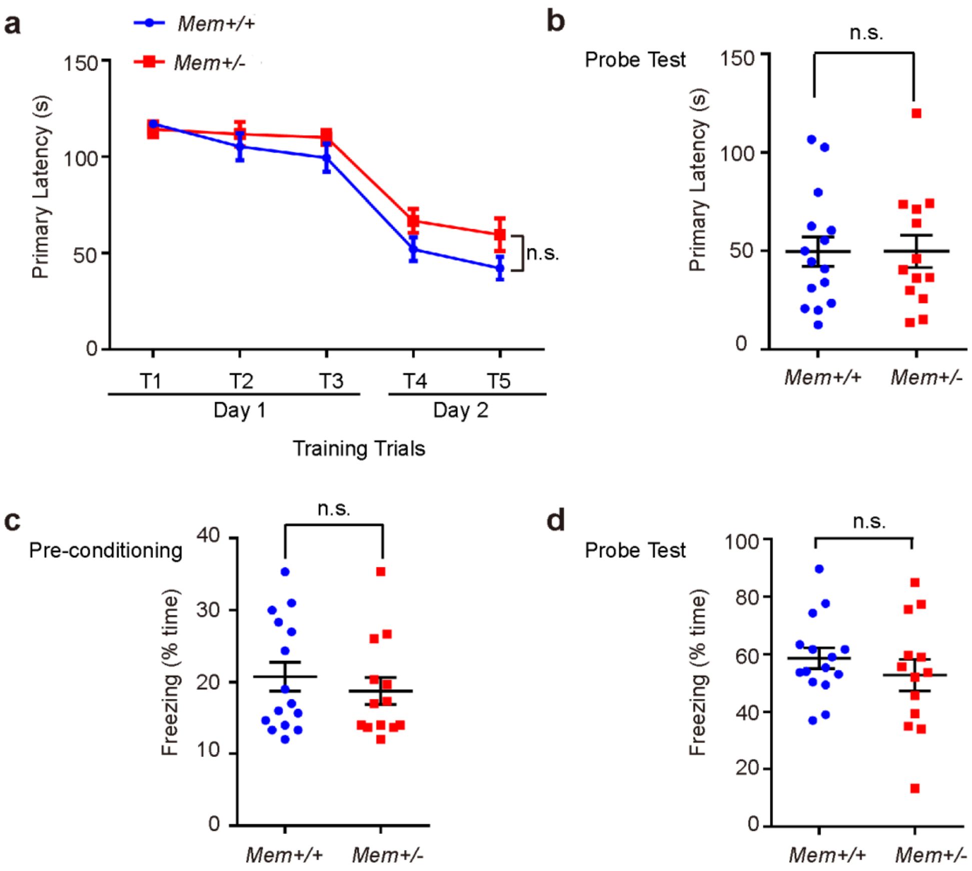

Supplementary Figure 9. Memory behavior tests in *Mem+/+* and *Mem+/-* mice (**a-b**) Barnes maze analysis: (**a**) Comparison of primary latency to target in *Mem+/+* or *Mem+/-* mice in five training trials (*n* = 15, *Mem+/+* and *n* = 13, *Mem*+/-). (**b**) Comparison of primary latency to target in *Mem+/+* or *Mem+/-* mice in probe tests (*n*= 15, *Mem+/+*, and *n* = 13, *Mem*+/-). (**c-d**) Fear conditioning analysis: (**c**) Comparison of freezing time (% time) in *Mem+/+* or *Mem+/-* mice (*n* = 15, *Mem+/+*, and *n* = 13, *Mem*+/-) during pre-conditioning. (**d**) Comparison of freezing time (% time) following electrical foot-shock in *Mem+/+* or *Mem+/-* mice (*n*= 15, mice for *Mem+/+*, and *n* = 13, mice for *Mem*+/-) during the probe test. Data represents mean ± s.e.m. unpaired t-test, n.s., not significant.

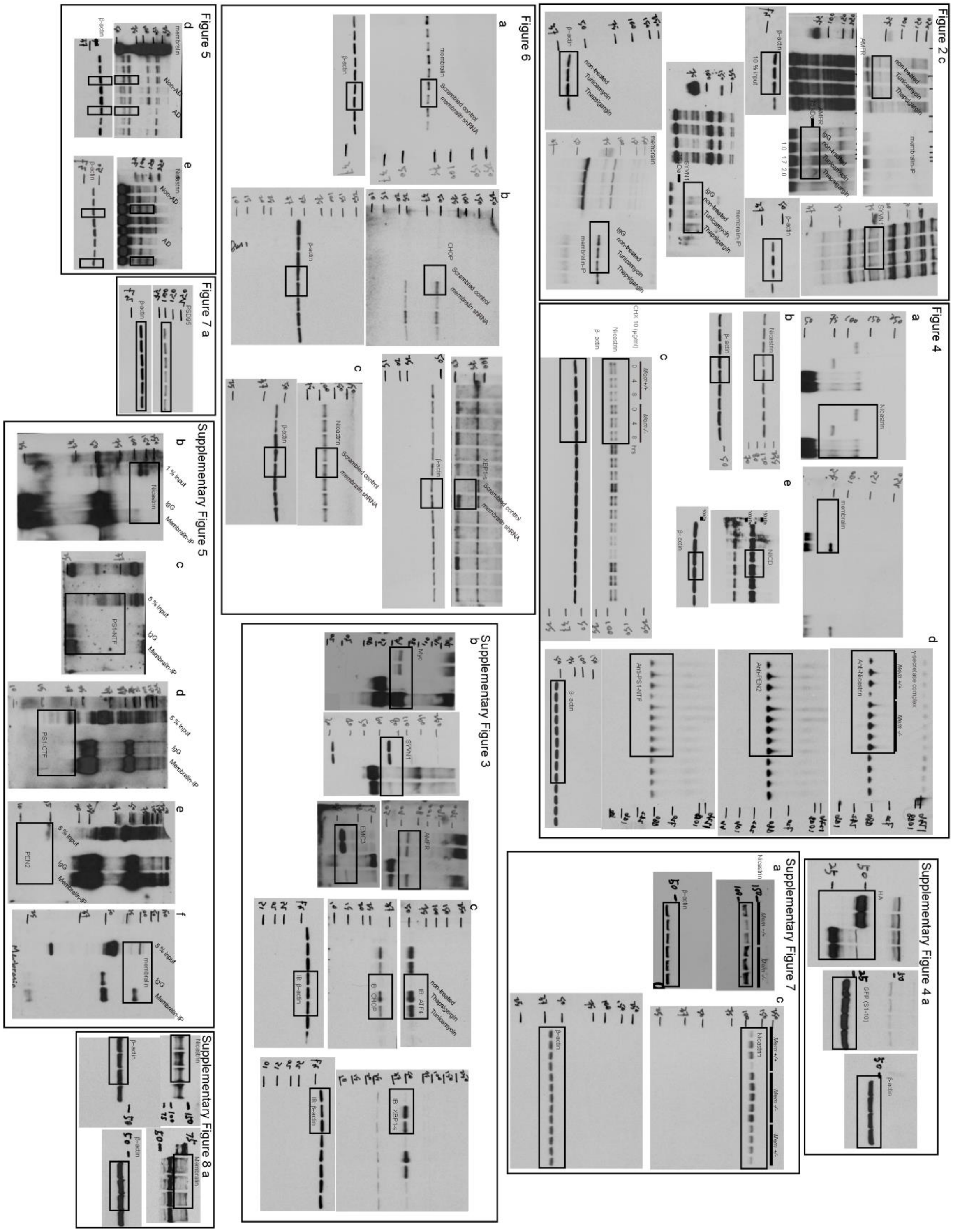

Supplementary Figure 10. Original western blot image scans corresponding to the figures Portions of the blots presented in the paper are indicated in the original scans as indicated by squared boxed regions.

Supplementary Table 1. List of membralin-interacting proteins detected using three different lysis buffers

96 common elements in Digitonin, NP40 and TritonX100		
TMEM33	ATP6AP1	GPAA1
ADAMTS1	LAMC1	PIGK
EMC3	DHRS7B	C3orf58
DSG2	MGST2	QPCTL
CLPTM1	TTC17	ANKLE2
DNAJC10	ATP6AP2	CPD
GPX8	PDLIM7	DAD1
EMC7	SEC11A	EGFL7
GJA1	FNDC3A	GOLPH3L
MAGT1	YIF1B	GPR180
OS9	TOR1AIP2	MLF2
FAM8A1	TIMP1	ORMDL3
CLGN	ACP2	SDF2
TMEM39A	METTL13	ATRAID
STC2	NGLY1	FBN2
UBAC2	CISD2	SCLY
RNF170	EXT2	SQSTM1
AUP1	MRPL23	TMEM161A
SEL1L	ATP13A1	ADAM9
CLPTM1L	CHID1	BRI3
HERPUD1	TMEM57	SLC7A1
PIGS	BOP1	CCSMST1
PIGT	DCAKD	GPR89
UBL4A	C1orf85	NOP56
RNF5	NAT14	TEX264
TBL2	SGCB	CEPT1
GALNT2	SLC38A9	TMEM115
GET4	GDF15	KDELC1
NDUFB8	NOC4L	LMF2
SARAF	PLOD2	NCSTN
AMFR	SEC63	PTDSS1
MGAT2	B3GNT1	TM9SF2

51 common elements in NP40 and TritonX100		
MBOAT7	SMPD4	EXOC2
DHCR24	HSDL1	EXOSC2
TMEM165	COX7C	EXOSC3
MARC2;MARC1	PDXDC1	IGF2R
SFXN4	STX5	LSM1
COX15	USP30	POLR3E
EI24	EARS2	PTPRF
ARL6IP1	PVR	SPNS1
TMEM97	SLC39A14	TMX4
DEGS1	TAMM41	TOR1A
ATG9A	TM2D3	VKORC1L1
ATP6V0A1	TMEM206	VPS51
GGCX	NDC1	YIPF5
NDUFS8	FUCA2	LCLAT1
PLD3	ADAM10	STX18
SCARB2	CLCN7	WLS
SLC30A7	ELOVL1	ORC4
16 common elements in Digitonin and NP40		
DNAJB12	TMEM179B	ALG1
CTU1	BNIP1	DNAJC11
ERLEC1	GTF2B	GEMIN2
FAM207A	MYO6	STIM1
LRPAP1	SELK	
MINA	TRABD	
5 common elements in Digitonin and TritonX100		
RTN3	MRTO4	
TRAPPC5	TXNDC15	
WBP11		
8 elements in Digitonin only		
TMUB2	VAPA	OGT
EMC8	METTL7A	TMEM214
EMC4	MRPL3	
4 elements in TritonX100 only		
LMNA	ATP6V1D	
WRNIP1	TPP1	

Supplementary Table 2. List of human brain tissues used for RNA analysis

Number	Source	Identifier	Diagnosis	Gender	Age	Braak
1	UCSD	X5709	normal	M	94	2
2	UCSD	X5628	normal	F	80	1
3	UCSD	X5248	normal	F	93	1
4	UCSD	X5114	normal	M	87	1.1
5	UCSD	X5070	normal	F	97	1
6	UCSD	X5049	normal	F	102	1
7	UCSD	X4954	normal	M	76	0
8	UCSD	X5783	normal	M	84	2
9	UCSD	X5302	normal	F	83	1.1
10	UCSD	X4996	normal	M	91	3
11	UCSD	X5795	AD	M	81	6
12	UCSD	X5789	AD	M	89	6
13	UCSD	X5732	AD	F	78	6.2
14	UCSD	X5707	AD	M	82	6.2
15	UCSD	X5693	AD	F	78	6.2
16	UCSD	X5691	AD	F	62	6.2
17	UCSD	X5686	AD	M	88	6.2
18	UCSD	X5685	AD	F	88	6.2
19	UCSD	X5680	AD	M	84	6.2
20	UCSD	X5788	AD	M	92	6
21	UCSD	X5697	AD	M	88	6.2
22	UCSD	X5720	AD	F	77	6.2

Supplementary Table 3. List of human brain tissues used for immunostaining and western blot

Number	Source	Identifier	Diagnosis	Gender	Age	Braak
1	UCSD	X5709	normal	M	94	2
2	UCSD	X5248	normal	F	93	1
3	UCSD	X5114	normal	M	87	1.1
4	UCSD	X5302	normal	F	83	1.1
5	UCSD	X5783	normal	M	84	2
6	UCSD	X5006	normal	M	69	0
7	UCSD	X5130	normal	M	71	1
8	UCSD	X4942	normal	M	83	0
9	UCSD	X5680	AD	M	84	6.2
10	UCSD	X5789	AD	M	89	6
11	UCSD	X5685	AD	F	88	6.2
12	UCSD	X5788	AD	M	92	6
13	UCSD	X5697	AD	M	88	6
14	UCSD	X5795	AD	M	81	6
15	UCSD	X5720	AD	F	77	6.2
16	UCSD	X5667	AD	F	71	6.2
17	UMiami	HBFF_16_004	AD	M	85	III-IV
